# A Review of Accidental Aerosol Generation in Laboratories and Laboratory-associated Infections

**DOI:** 10.1089/apb.2024.0014

**Published:** 2025-08-29

**Authors:** Ashley R. Ravnholdt, Shanna A. Ratnesar-Shumate, Joshua L. Santarpia

**Affiliations:** ^1^ Global Center for Health Security, University of Nebraska Medical Center, Omaha, Nebraska, USA.; ^2^ Department of Pathology and Microbiology, University of Nebraska Medical Center, Omaha, Nebraska, USA.; ^3^ National Strategic Research Institute, Omaha, Nebraska, USA.

**Keywords:** laboratory-acquired infection (LAI), laboratory aerosol generation, accidental aerosol generation, biosafety, biological laboratory safety

## Abstract

**Introduction:**

Laboratory-acquired infections (LAIs) from exposure to infectious biological pathogens during laboratory operations present ongoing challenges despite modern biosafety measures. Notably, LAIs attributed to inhaling infectious aerosols continue to occur.

**Objective:**

This review aims to enhance understanding of the risks of LAIs associated with infectious aerosols. The first objective of this review is to summarize studies that have characterized the potential for accidental aerosol generation in biological laboratories and to synthesize these findings. The second objective is to examine past LAI incidents involving infectious aerosol exposure to identify knowledge gaps.

**Methods:**

A literature review using PubMed and Google Scholar databases with relevant keywords was performed. The results were screened to identify studies that reported LAIs related to aerosol exposures. Thirty-eight articles pertaining to LAIs involving infectious aerosol exposures from an initial pool of 63 articles were identified.

**Discussion and Conclusions:**

This review underscores the need for comprehensive safety protocols to mitigate risks associated with LAIs due to aerosol generation. It identifies critical gaps in understanding aerosol dynamics, such as particle size distribution and the influence of laboratory automation. The review also highlights the inadequacy of current LAI reporting practices, advocating for improved documentation and standardized methods to enhance biosafety in laboratory settings.

## Introduction

Infections derived from accidental exposure to pathogens following laboratory-related activities or accidents are termed “laboratory-acquired infections,” “laboratory-associated infections,” or LAIs.^1^ These infections can result from exposures to bacterial, viral, fungal, or parasitic pathogens; all of which can be transmitted through various exposure routes. These routes include percutaneous inoculations like needle pricks, direct contact between contaminated surfaces and mucosal membranes, ingestion from unsafe laboratory practices such as mouth pipetting, and inhaling infectious aerosols. The consequences of LAIs vary depending on the pathogen and the host individual, potentially resulting in conditions ranging from asymptomatic infections to acute, chronic, or even fatal diseases. This review underscores the significant yet underexplored risk of accidental infectious aerosol generation in biological laboratories, highlighting the critical knowledge gaps where specific aerosol-generating procedures (AGPs) often remain unidentified.

The history of LAIs in the United States dates back to 1885 with the first recorded LAI case of typhoid fever^2^; however, LAIs were not widely tracked, recorded, or reported until the 1950s.^3,4^ Following these reports, the Biological Safety Cabinet (BSC) development in 1958 marked a pivotal advancement in containing pathogens to prevent future LAIs.^5^ Adopting general biosafety practices, including using BSCs, personal protective equipment, and laboratory personnel training on proper biological handling techniques, significantly reduced LAI occurrences post-1950s.^5^ Despite these advances, LAIs continue to occur, with several incidents involving biological select agents reported between 2004 and 2010.^6^ These incidents highlight a persistent challenge in biosafety: identifying the specific laboratory procedures responsible for infectious aerosol generation implicated in these reports.^6^

Determining the cause of an inhalation derived LAI presents unique challenges, as it is not visible or immediately noticeable as other transmission routes such as a needle prick. While inhalation is a well-recognized transmission route, particularly evident when laboratory workers contract infections without direct handling of the causative pathogen,^5^ it is often under-characterized. Reports of LAIs frequently imply aerosol exposure but do not conclusively identify the aerosol sources. This review calls for a focused examination of aerosols related to LAIs to address this complex transmission mechanism and enhance the overall understanding and management of these risks.

Many laboratory procedures are thought to generate aerosols, inadvertently increasing the risk of pathogen exposure. Recognized AGPs include pipetting, shaking, mixing, and other procedures such as centrifugation and sonication.^7^ The Biosafety in Microbiological and Biomedical Laboratories (BMBL) manual provides guidelines for the safe handling and containment of biological agents and toxins, offering standards to prevent exposure and minimize risks in laboratory settings.^7^ While some of the BMBL’s guidelines for AGPs are informed by empirical data, expert consensus, and best practices, there remains a significant gap in empirical evidence supporting these guidelines.^7,8^ A thorough characterization of inadvertent aerosol generation within biological laboratories is needed to mitigate risks associated with infectious aerosol exposure effectively.^9^

Research on characterizing unintentional aerosol releases in laboratories is crucial for assessing the risks associated with certain laboratory procedures and accidents. It is essential to integrate detailed accounts of past LAIs linked to aerosol exposures to understand laboratory aerosol risks comprehensively. Such a comprehensive approach enhances the identification of pathogens frequently involved in LAIs and highlights the specific procedures and accidents leading to infectious aerosol generation, thereby informing the development of more effective biosafety measures and protocols.

The primary objective of this review is to synthesize findings from previous studies on inadvertent aerosol generation in laboratories and LAI incident reports to enhance our understanding of aerosol-related LAIs. By highlighting connections between aerosol generation in laboratory studies and LAI incidents, assessing the impact of personnel experience, and emphasizing the need for robust aerosol containment measures, this review aims to identify critical methodological gaps and deficiencies in laboratory studies and LAI reporting. These insights will inform the development of more precise biosafety measures and encourage implementing standardized reporting to improve laboratory safety protocols effectively.

## Methods

A search was conducted using PubMed and Google Scholar using the keywords “laboratory-acquired + infection,” “laboratory-associated + infection,” and “laboratory + aerosol + generation”. The resulting articles were screened to determine if the studies or reports were LAIs related to inadvertent aerosol exposure or generation studies, with the criterion that the articles must be offered in English. Reports were limited to LAIs involving bacterial and viral pathogens. The bibliographies of accepted articles were also screened for additional relevant studies and reports. Reports documenting incidents outside the United States were accepted, provided they were offered in English. A total of 63 articles were identified, and 38 were determined to fit this criterion and included in this review.

## Results

### Approach and Findings from Studies on Inadvertent Laboratory Aerosol Generation

Early foundational studies, such as those by Reitman and Wedum, used sieve-type aerosol samplers to capture and analyze aerosols generated during varied laboratory procedures, including blending, centrifuging, decanting, inoculating, lyophilizing, pipetting, and opening vials containing liquid cultures.^4^ This study revealed that certain procedures, notably blending, decanting, centrifuging, and pipetting, led to high microbial aerosol generation, indicated by substantial colony counts on agar plates.^4^

Several studies focused on quantifying microbial aerosol concentrations, measuring the number of microorganisms per cubic meter of air (CFU/m^3^). For instance, Tomlinson (1957) utilized a slit aerosol sampler to monitor microbial concentrations over time after opening screw-capped bottles.^10^ They observed the highest microbial aerosol concentration, 15.0 CFU/m^3^, occurring 4–6 min after opening the bottles.^10^ The concentration during the opening was slightly lower at 8.8 CFU/m^3^, and the lowest concentration, 7.5 CFU/m^3^, was recorded 5–7 min post-opening.^10^ These data highlight the temporal dynamics of aerosol persistence, underlining the importance of monitoring aerosols post-generation to assess exposure risks thoroughly.^10^

In a study by Pottage et al., microbial aerosol concentrations were quantified during serial dilutions and plating procedures, focusing on the influence of operator experience on aerosol generation.^11^ The findings indicated that novice scientists generated higher aerosol concentrations, averaging 14.7 CFU/m^3^, compared with their more experienced counterparts, who averaged 9.9 CFU/m^3^.^11^ These data underscore the importance of proper training and technique in minimizing the risks associated with aerosol generation.^11^

Further research by Pottage et al. examined the variability in aerosol production across different techniques and pathogen concentrations.^12^ This study involved routine microbiological procedures considered to be performed with proper technique (e.g., pipette mixing, serial diluting, vortex mixing, and plating).^12^ They also simulated poor techniques or accidental scenarios (e.g., flicking open an Eppendorf tube or dropping a tube).^12^ Notably, the highest microbial concentrations were observed with improper techniques, with flipping open Eppendorf snap-top tubes resulting in the highest recorded microbial concentration of 8000 CFU/m^3^.^12^ The findings concluded that both technique and pathogen concentration significantly impacted the generation of microbial aerosols.^12^

The work by Kenny and Sable, using an Andersen cascade impactor to analyze aerosol size distribution, provides critical insights into aerosol behavior in the laboratory environment.^13^ They measured the count median diameters (CMDs), identifying the diameter where half the particles in the sample are larger and half are smaller, thus offering a key metric of particle size distribution.^13^ For instance, using a laboratory blender with the lid on during operation and then removing it post-operation resulted in CMDs of <2 µm.^13^ Other activities such as harvesting an infected egg, mixing culture with pipettes or vortex mixers, using a centrifuge with spilled culture, sonic oscillation, dropping an infected egg or flask culture, or spilling culture from a pipette, produced CMDs ranging from 2 to 5 µm.^13^ Opening or dropping lyophilized cultures led to CMDs in the 8–10 µm range.^13^ They also noted that specific procedures like removing the lid from a blender, dropping lyophilized cultures, or dropping a flask containing culture, led to aerosol concentrations exceeding 1000 CFU/m^3^, highlighting the mishaps that pose significant aerosol risks.^13^

The study by Bennett and Parks utilized a cyclone sampler, a Casella slit sampler, and an Andersen sampler to quantify and characterize aerosols generated from simulated laboratory accidents within a controlled environment.^14^ They identified aerosol releases during accidents such as dropping, spilling, and mishandling laboratory equipment and liquids.^14^ They noted consistent aerosol concentration patterns between the Casella and Andersen samplers, though they found higher concentrations with the cyclone sampler.^14^ Notable high concentrations were observed during incidents involving dropping fungal plates, operating a centrifuge with a spill, dropping a large bottle, and syringe filter blockages.^14^ In addition, incidents like small and large spills, syringe filter blockages, dropping bottles or fungal plates, and centrifuge bucket accidents tended to produce the smallest aerosol particles, primarily those smaller than 2.2 µm in diameter.^14^

In a different approach, Miller et al. used surrogate markers, such as Dulbecco’s modified Eagle medium (DMEM), to assess inadvertent aerosol generation, thereby avoiding the use of microbial cultures.^15^ Their analysis with a digital particle counter showed that homogenizing DMEM generated particles predominantly within 0.3–5 µm in diameter, most commonly at 0.3 µm.^15^ This study highlights the variability of particle sizes produced by different laboratory procedures; for instance, vortexing yielded particles between 0.5 and 2 µm, whereas pipetting resulted in a broader range, from 0.5 to 10 µm.^15^ These findings underscore the importance of understanding particle size distributions in assessing aerosol risks associated with various laboratory procedures.^15^

A summary of aerosol characteristics measured from studies on inadvertent laboratory aerosol generation is provided below in Table 1. The table provides the aerosol properties assessed in each study, including microbial aerosol concentration, size, and colony count (only). The term “Colony count (only)” pertains to the methodology employed in the 1956 study by Reitman and Wedum, where the volume of air passed through the sampler was not noted, thereby restricting the microbial aerosol concentration from their findings.^4^ The table also differentiates the type of test aerosol used in each study as nonbiological or biological surrogates.

**Table 1.** Summary of aerosol-related LAI studies

	Measured aerosol characteristics			Test aerosol surrogate	
Study	Microbial aerosol concentration	Size	Colony count (only)	Nonbiological	Biological
Bennett and Parks^14^	X	X			X
Kenny and Sabel^13^	X	X			X
Miller et al.^15^		X		X	
Pottage et al.^11^	X			X	X
Pottage et al.^12^	X			X	X
Reitman and Wedum^4^			X		X
Tomlinson^10^	X				X

This table provides an overview of the aerosol characteristics assessed in past aerosol-related LAI studies, allowing for a comparative analysis of the methodology across different research efforts. By separating data based on the type of test aerosol and aerosol properties studied, this table highlights the diverse approaches taken to understand inadvertent aerosol generation in laboratories from prior studies. X indicates that the study included the corresponding characteristics or test aerosol.

### Past LAI Incident Reports

Building on the previous section that detailed studies on accidental aerosol generation in laboratories, this section aims to provide real-world context by summarizing specific reports on LAIs related to aerosols, organized by pathogen type as outlined in Table 2. This analysis of aerosol-related LAI reports has identified specific incidents involving a broad range of pathogens, including 10 bacterial and 13 viral types. These incidents highlight the diverse laboratory procedures that have led to LAIs, ranging from routine procedures to accidents like dropping flasks or mishandling equipment.^16–39^

**Table 2.** Summary of previously reported aerosol-related LAI by pathogen type

Pathogen	Number of aerosol-related LAIs identified	Incident description	Potential source of inadvertent aerosol generation	Reference
*Bacillus anthracis*	1	An instance of LAI, followed by an anthrax outbreak in the neighboring community, occurred in the former Soviet Union. This outbreak was associated with a supposed inadvertent release of *B. anthracis* spores from a research facility, an event referred to in the literature as the Sverdlovsk anthrax leak.	Nonspecific laboratory procedures	^16,17^
*Brucella melitensis*	3	A researcher dropped a flask. Nonspecific laboratory procedures on a laboratory bench outside of a BSC.	Spilling a blood culture; nonspecific laboratory procedures on an open bench	^18^
*Brucella spp.*	59	Various laboratory procedures such as subculturing, Gram staining, sniffing plates, and other procedures completed on an open bench were noted.	Various laboratory procedures (e.g., subculturing, Gram staining, sniffing plates); nonspecific laboratory procedures on an open bench	^19^
*Burkholderia mallei*	6	A researcher accidentally dropped a flask onto the laboratory floor. The researcher who dropped the flask and a researcher present in the laboratory both fell ill. Two researchers fell ill after completing protocols for harvesting bacteria which involved washing plates outside of a BSC. Two researchers fell ill after aerating cultures, and it was noted that the containers were opened immediately following aeration.	Dropping a flask; various laboratory procedures (e.g., washing plates, opening vials following aeration)	^20^
*Burkholderia pseudomallei*	4	A researcher sonicated a large, unspecified volume of culture outside a BSC. Three cases of melioidosis occurred following nonspecific aerosol exposures, with one infection occurring in a laboratory employee who never directly handled *B. pseudomallei.*	Sonicating; nonspecific laboratory procedures	^21,22^
Dengue virus	2	A researcher filtered viral samples through a nonspecific filtering mechanism during an isolation procedure. A researcher fell ill after following procedures from nonspecific viral propagation and purification protocols.	Filtering; nonspecific laboratory procedures from viral propagation and purification protocols	^23,24^
*Escherichia coli* (strain O157:H7)	4	Two researchers fell ill after vortexing cultures on open benchtops. Two researchers fell ill after completing latex agglutination tests, which typically involve shaking samples.	Various laboratory procedures for mixing (e.g., vortex, shaking by hand)	^25^
*Francisella tularensis*	1	A tularemia case occurred in a laboratory technician that never directly handled *F. tularensis*, but cultures were handled in their laboratory.	Nonspecific laboratory procedures	^16^
Hantavirus	2	Two researchers fell ill after operating a homogenizer, one infection occurred in the operator, and the other occurred in a researcher who worked in the same facility but never directly handled specimens.	Operating a homogenizer	^26^
Lymphocytic choriomeningitis virus		Laboratory workers fell ill after completing nonspecific routine laboratory procedures with contaminated cell cultures.	Nonspecific laboratory procedures with cell cultures	^27^
Mayaro virus	1	The researcher operated a malfunctioning vacuum pump containing Mayaro viral antigens within a laminar flow hood.	Vacuum pump malfunction	^28^
*Neisseria meningitidis*	17	A microbiologist fell ill after handling cultures outside a BSC; 16 researchers fell ill after completing nonspecific laboratory procedures.	Open bench work; nonspecific laboratory procedures	^29,30^
Parvovirus B19	9	Nine employees fell ill after completing nonspecific laboratory procedures. Only two of the employees directly handled the virus.	Nonspecific laboratory procedures	^22,31^
Rabies virus	3	A researcher fell ill after operating a homogenizer. Researchers completed nonspecific laboratory procedures.	Operating a homogenizer; nonspecific laboratory procedures	^16,22^
Sabiá virus	1	Following the operation, a researcher cleaned a minor spill observed within a centrifuge.	Opening a centrifuge after operation that contains a spill	^32^
SARS-associated coronavirus	1	A student fell ill after completing nonspecific routine laboratory procedures with a contaminated cell line.	Nonspecific laboratory procedures with cell cultures	^33^
Tick-borne encephalitis virus	2	A researcher fell ill after an unspecified accident occurred. A researcher fell ill after completing a viral isolation procedure predominantly conducted inside a BSC, except for a centrifugation step.	Nonspecific laboratory procedures (e.g., viral isolation, centrifugation)	^16,34^
Vaccinia virus	2	A researcher transferred a covered 96-well plate to a plate reader outside of a BSC, where the plate lid was removed during operation. Researcher performed routine procedures with cell cultures containing high virus concentrations.	Plate reader moving the uncovered plate during operation. Nonspecific laboratory procedures with cell cultures	^35,36^
Venezuelan equine encephalitis virus	1	Although the researcher who fell ill never directly handled the virus, they worked in a laboratory adjacent to a laboratory that was producing a high-titer viral stock.	Nonspecific laboratory procedures	^28^
*Vibrio cholerae*	1	A researcher fell ill after cleaning a spill from a culture flask that toppled over while left on a running shaker.	Spilling liquid cultures	^37^
Western equine encephalitis virus	1	A centrifuge accident occurred, releasing visible droplets of viral culture around the laboratory.	Centrifuge accident	^28^
*Yersinia pestis*	2	Two researchers fell ill after nonspecific laboratory procedures led to aerosol generation.	Nonspecific laboratory procedures	^38^
Zika virus	2	Two researchers fell ill after decanting a viral culture into a funnel within a BSC, suggesting a splash outside the BSC.	Decanting	^39^

This table contains LAI reports broken down by pathogen type, number of aerosol-related LAIs identified, incident description, and the potential source of inadvertent aerosol generation, if provided. These LAI reports were identified during the literature search, as mentioned in the methods. This table represents all reports accepted from the literature search.

From the 24 accepted reports, a total of 125 LAIs were identified, with bacterial pathogens causing 98 and viral pathogens causing 27 LAIs. The most common source of inadvertent aerosol generation implicated in these LAI reports was associated with vague or nonspecific laboratory procedures.^16–19,21,22,24,27,28,30,31,33,34,36,38^ Specifically, 85 bacterial LAIs and 16 viral LAIs were linked to or suggested to involve nonspecific laboratory procedures. Notably, infections involving *Brucella* species accounted for the highest number of LAIs attributed to a single pathogen type, with 62 cases often arising from open bench procedures such as subculturing, Gram staining, and plate sniffing.^18,19^

Several incidents involving laboratory equipment have been directly linked to LAIs. Notable equipment failures include centrifuge mishaps that dispersed Sabiá and western equine encephalitis viruses,^28,32^ and a vacuum pump malfunction contributed to the dispersal of Mayaro virus.^28^ Beyond equipment mishaps, routine use of certain laboratory instruments, such as homogenizers and sonicators, has also been associated with LAIs. Specifically, aerosols containing hantavirus and rabies virus were generated during homogenization procedures.^16,26^ Similarly, sonication was suspected to aerosolize *Burkholderia pseudomallei*.^22^ In addition, the use of plate readers in handling vaccinia virus,^35^ along with practices like vortexing *Escherichia coli* on an open bench,^25^ and decanting Zika virus,^39^ have been identified as key sources of inadvertent aerosol generation in LAI reports.

Accidental spills or drops by researchers were frequently cited as sources of unintentional aerosol generation, resulting in LAIs. Such incidents include mishaps where cultures of *Vibrio cholerae* and Sabia virus were spilled, subsequently leading to LAIs.^32,37^ Similarly, incidents involving dropped cultures of *Brucella melitensis* and *Burkholderia mallei* were documented prior to LAIs.^18,20^

The broad range of pathogens involved and the diverse laboratory activities linked to these incidents highlight the critical need for further research into accidental aerosol generation and transmission mechanisms in laboratories. Such research is needed to enhance the understanding of LAI risks and developing more effective measures to prevent exposure. The challenge of accurately identifying the sources of aerosol generation further underscores the difficulties in tracking and mitigating exposure risks.

## Discussion

This review of inadvertent aerosol generation in laboratories offers substantial insights into aerosol-related LAIs dynamics. It reveals the connection between aerosol generation in laboratory studies and LAI reports, highlights the influence of personnel experience, and emphasizes the importance of implementing aerosol containment. In addition, the review has identified critical methodological gaps in previous studies, laying the groundwork for future research to assess accidental infectious aerosol generation in laboratories. These gaps include particle size distribution, temporal aerosol patterns, environmental conditions, highly automated equipment, and an integrative and standardized sampling approach. Furthermore, the review highlights the deficiencies of past LAI reporting, which often lacks detailed procedural recounts or is underreported. These shortages complicate efforts to assess and mitigate risk effectively. This inadequate reporting hinders the development of targeted safety protocols and underscores the need for improved reporting systems to better understand and prevent LAIs.

A connection between laboratory studies and LAI reports emerges with certain procedures posing increased aerosol risks. Vortex mixing, decanting, homogenizing, and sonicating microbial materials have been shown to generate aerosols in controlled laboratory studies^4,12,13,15^ and were implicated in real-world LAI incidents.^16,22,25,26,39^ In addition to laboratory procedures, frequent laboratory accidents such as drops, obstructions (e.g., pump and syringe blocks), and spills frequently result in infectious aerosol generation in controlled laboratory studies^12–14^ and have been implicated in real-world LAI incidents.^18,20,28,32,37^

Pottage et al. demonstrated that aerosol generation can vary significantly between operators performing the same procedure.^11^ They observed a statistically significant difference in microbial aerosol concentrations when novice and experienced personnel conducted serial dilutions.^11^ This variation suggests that operator experience influences aerosol generation,^11^ emphasizing the importance of comprehensive and targeted biosafety training to minimize the risk of LAIs by equipping personnel with the necessary skills to handle potentially hazardous situations safely.^8^

Controlled laboratory studies demonstrate that numerous procedures conducted in biological laboratories can generate potentially infectious aerosols.^4,10–14^ Prioritizing aerosol containment with established best practices, such as BSCs and other secondary containment, is needed to maintain safety.^7^ By analyzing past LAI incidents and integrating aerosol generation data into biosafety guidelines, we can further reduce aerosol-related risks associated with LAIs. Enhancing biosafety protects laboratory personnel and serves the public by minimizing the potential spread of infectious aerosols.^40^

While enhancing biosafety measures is crucial to controlling aerosol risks, a thorough review of past laboratory studies on inadvertent aerosol generation has highlighted several methodological gaps that must be addressed to advance our understanding and control of aerosol risks. A prevalent methodological gap identified in the reviewed studies is calculating aerosol size distribution, which is the range and proportion of particle sizes within a sample.^41^ Although seven studies provided quantitative measurements of aerosol generation resulting from simulated laboratory activities and accidents,^4,10–15^ focusing primarily on microbial concentration or particle counts, six of these studies omitted aerosol size distribution. Understanding the aerosol size distribution is crucial for understanding aerosol transport, deposition in the respiratory tract, and stability.^41^

Assessing the stability of infectious aerosols post-procedure is a crucial aspect of understanding laboratory aerosol dynamics. Without accounting for these temporal dynamics, reports on microbial aerosol concentrations may inaccurately represent the duration of risk following an accidental aerosol release. Bioaerosol stability inherently depends on environmental conditions^42^; therefore, monitoring conditions such as relative humidity, ultraviolet radiation, and temperature is a critical methodological aspect that has been overlooked in the studies reviewed. Aerostability varies from pathogen to pathogen, as do other properties that may influence the likelihood of aerosol production (e.g., hydrophilicity or hydrophobicity^43^).^44^ Studies using pathogens or studies with surrogates that can accurately mimic pathogen properties are required to characterize risk fully.^45^

Another significant area requiring attention is the characterization of aerosol generation from highly automated laboratory equipment, such as plate washers. While the studies reviewed typically investigate common laboratory procedures and accidents like pipetting, mixing, vial opening, plate dropping, and centrifuge spills, the increasing use of automated equipment introduces new potential sources of inadvertent aerosol generation. This shift necessitates an expanded focus on research to understand how automation influences aerosol dynamics and identify any risks associated with these technologies. As laboratories continue to integrate automation with infectious material, it is crucial to assess how these systems contribute to aerosol production and to develop specific mitigation strategies tailored to automated processes.

Understanding aerosol behavior in laboratory environments requires an analysis of complex factors that influence the dynamics of aerosol particles, including their physical state, stability, movement, and potential for pathogen transmission and infection.^46^ Accurate risk assessment and effective biosafety measures hinge on a deep understanding of these dynamics, necessitating an integrative approach that uses multiple instruments in tandem to assess inadvertent aerosol production in laboratories. Different aerosol instruments focus on various aerosol properties: for instance, cascade impactors for time-integrated measurements of size-resolved mass, optical particle counters for physical size distributions, and specialized collection samplers like impingers to assess viability.^41^ This tiered approach to aerosol sampling, leveraging diverse samplers to capture different particle characteristics and biological potentials, is crucial for effectively understanding and managing aerosol risks in laboratories, supporting the development of robust biosafety protocols.

A standardized analysis approach that considers instrument properties (e.g., collection efficiency and sampler flow rate) is needed to ensure consistency and comparability of data across different studies and equipment.^47^ Bennett and Parks (2006) revealed the need for standardized methods, which noted variations in microbial aerosol concentration when using different types of samplers during the same experiments.^14^ Similarly, variations in microbial aerosol concentration and colony counts across the studies presented here may result from differences in collection efficiency, limitations of the sampling methods, or the lack of value normalization across similar procedures and simulated accidents. This highlights the need for a unified methodology to assess and compare aerosol generation in laboratory settings accurately.

The current literature often needs more detail to effectively determine the aerosol source that led to LAIs, which is critical for informing biosafety measures. Substantial policy changes addressing incident reports are needed to understand the root causes of aerosol-related LAIs and enhance laboratory safety. This review highlights significant deficiencies in current reporting practices, including the need for more detailed procedural recounts and a commitment to encouraging LAI reporting.

A significant challenge in understanding LAIs related to aerosol generation lies in the frequent citation of “nonspecific laboratory procedures” in incident reports.^16–19,21,22,24,27,28,30,31,33,34,36,38^ Although initially seeming less informative, their recurrent mention highlights a critical knowledge gap. This pattern highlights the need for a standardized approach to documenting LAI incidents, which needs improvement in the biosafety field. The absence of detailed procedural information hinders identifying specific activities leading to aerosol generation and limits our ability to develop targeted preventive measures. Also, it indicates a need for enhanced surveillance and reporting systems to better understand and mitigate the risks associated with routine laboratory activities. Importantly, this gap drives the need to characterize the aerosol generation potential of individual laboratory procedures to ensure precise and effective control measures.

A persistent issue, as highlighted by Reitman and Wedum, is the widespread reluctance to report LAIs,^4^ often due to concerns about the negative reputation of researchers and their laboratories. To address this, implementing a policy to mandate anonymous reporting of LAI incidents could foster more detailed disclosures. A shift toward more detailed reporting is needed for accurate analysis and improvement of laboratory preventative measures. Currently, the literature relies on voluntary LAI reports, with no global mandate for systematic reporting of LAIs caused by pathogens not classified as U.S. biological select agents and toxins. Providing more detailed accounts of procedures or general protocols leading up to LAI incidents would enable more effective analysis and help improve preventive measures. Reports have indicated that routine activities, such as plate transfers, are sources of infectious aerosol releases, emphasizing the need for further research into the aerosol-generating potential of everyday laboratory tasks.

## Conclusions

This review has highlighted the importance of several areas in understanding LAIs due to inadvertent aerosol generation. First, operator experience is crucial; enhanced training and robust aerosol containment strategies are essential to minimize risks. Proper training equips laboratory personnel to implement these strategies, effectively reducing aerosol-related LAIs.

Second, the review identifies key methodological gaps needing attention: (1) aerosol size distribution, (2) bioaerosol stability, (3) aerosol persistence under various environmental conditions, (4) the need for integrative sampling approaches, (5) standardized analysis to normalize results, and (6) evaluation of aerosol generation from highly automated lab equipment. Addressing these gaps will lead to more accurate risk assessments and the development of more effective prevention and containment strategies.

Furthermore, deficiencies in the current LAI reporting system, characterized by vague details and a culture of underreporting, impede adequate understanding and prevention of LAIs. The review advocates for policy changes encouraging detailed and mandatory anonymous reporting of LAIs. By addressing these areas, future research can enhance biosafety in laboratories, safeguarding laboratory personnel and the public from the risks of inadvertent aerosol generation and the potential spread of infections.
